# Strengthening malaria service delivery through supportive supervision and community mobilization in an endemic Indian setting: an evaluation of nested delivery models

**DOI:** 10.1186/1475-2875-13-482

**Published:** 2014-12-08

**Authors:** Ashis Das, Jed Friedman, Eeshani Kandpal, Gandham N V Ramana, Rudra Kumar Das Gupta, Madan M Pradhan, Ramesh Govindaraj

**Affiliations:** Health, Nutrition and Population, The World Bank, 1818 H St NW, Washington, DC 20433 USA; Development Research Group, The World Bank, 1818 H St NW, Washington, DC 20433 USA; Health, Nutrition and Population, The World Bank, Delta Center, Menengai Rd, Upper Hill, Nairobi, Kenya; National Vector Borne Disease Control Programme, 22, Shamnath Marg, New Delhi, India; Department of Health and Family Welfare, Bhubaneswar, India; Health, Nutrition and Population, The World Bank, 70 Lodi Estate, New Delhi, India

**Keywords:** Community health worker, Supportive supervision, Community mobilization, Malaria, India

## Abstract

**Background:**

Malaria continues to be a prominent global public health challenge. This study tested the effectiveness of two service delivery models for reducing the malaria burden, e.g. supportive supervision of community health workers (CHW) and community mobilization in promoting appropriate health-seeking behaviour for febrile illnesses in Odisha, India.

**Methods:**

The study population comprised 120 villages from two purposively chosen malaria-endemic districts, with 40 villages randomly assigned to each of the two treatment arms, one with both supportive supervision and community mobilization and one with community mobilization alone, as well as an observational control arm. Outcome measures included changes in the utilization of bed nets and timely care-seeking for fever from a trained provider compared to the control group. Analysis was by intention-to-treat.

**Results:**

Significant improvements were observed in the reported utilization of bed nets in both intervention arms (84.5% in arm A and 82.4% in arm B *versus* 78.6% in the control arm; p < 0.001). While overall rates of treatment-seeking were equal across study arms, treatment-seeking from a CHW was higher in both intervention arms (28%; p = 0.005 and 27.6%; p = 0.007) than in the control arm (19.2%). Fever cases were significantly more likely to visit a CHW and receive a timely diagnosis of fever in the combined interventions arm than in the control arm (82.1% vs. 67.1%; p = 0.025). Care-seeking from trained providers also increased with a substitution away from untrained providers. Further, fever cases from the combined interventions arm (60.6%; p = 0.004) and the community mobilization arm (59.3%; p = 0.012) were more likely to have received treatment from a skilled provider within 24 hours than fever cases from the control arm (50.1%). In particular, women from the combined interventions arm were more likely to have received timely treatment from a skilled provider (61.6% vs. 47.2%; p = 0.028).

**Conclusion:**

A community-based intervention combining the supportive supervision of community health workers with intensive community mobilization and can be effective in improving care-seeking and preventive behaviour and may be used to strengthen the national malaria control programme.

**Electronic supplementary material:**

The online version of this article (doi:10.1186/1475-2875-13-482) contains supplementary material, which is available to authorized users.

## Background

Globally, malaria control programmes have experimented with innovative strategies aligned with the healthcare delivery system status of each country [1]. One of the foremost strategies involves the introduction of community-based management of malaria through the deployment of community health workers [2–6]. During the last decade, India’s malaria control strategies under the aegis of the National Vector Borne Disease Control Programme (NVBDCP) introduced this strategy among other innovations to strengthen its fight against malaria [7] as the disease burden remains high – India continues to contribute around two-thirds of confirmed malaria cases in the South East Asia region of the World Health Organization [8]. The endemic eastern and central regions of the country, in particular, experience adverse socio-economic impacts due to their malaria burden [7, 9].

Under the community-based approach, the village CHW, known as Accredited Social Health Activist (ASHA) is designated to address early detection, management and prevention of malaria at the community level [7, 10, 11]. The ASHAs have been trained to test for *Plasmodium falciparum* malaria cases using rapid diagnostic tests and to treat these cases with artemisinin combination therapy (ACT). To further prevent any delays in the diagnosis or treatment of malaria, the ASHAs have also been provided with the requisite supplies of Rapid Diagnostic Test (RDT) kits and ACT [7, 10]. In addition, long-lasting insecticide-treated bed nets (LLIN) have been distributed free of cost to populations in high endemic districts to strengthen prevention activities [7].

The global evidence on malaria management suggests necessary preconditions to ensure the effectiveness of community-based approaches [12]. For instance, the community should engage at the inception and planning stage rather than being mere recipients. Developing intervention modalities at the community level through institutions and individuals further enhances the community's participation and ownership. Communities should be empowered to regularly monitor and evaluate the effectiveness of interventions [8]. In terms of the involvement of CHW, the global evidence suggests that regular and systematic supervision with clearly defined objectives can improve the performance of community health workers involved in primary health care [13–16]. Such evidence for India, however, is lacking and insufficient community capacity, trust, and coordination may keep the new malaria control strategies from meeting expected outcomes [9, 17, 18]. Hence, without addressing these community level impediments, ongoing control efforts may lead to diminished outcomes and the wastage of resources.

This study tests the effect of two complementary community-based interventions implemented in Odisha state, India, through local non-government organizations (NGO) to support NVBDCP’s ongoing efforts. The two interventions, in essence a partnership between the public sector, the private sector and the community, tested the effectiveness of:

- community mobilization promoting appropriate malaria related behaviour, such as bed net use and timely and appropriate care-seeking from a community level designated provider (i.e. CHW) for febrile illnesses
- supportive supervision of community health workers (CHW) on effective malaria case management

These interventions provide evidence not only on effectiveness but also possible scale up to similar settings. More generally, the findings should inform the development of a pragmatic policy approach to malaria control.

## Methods

### Study settings

This study was carried out in Mayurbhanj and Sundargarh districts of Odisha. These areas are characterized by scheduled tribe (indigenous) populations and hilly and forest habitations [19, 20]. The districts were purposively selected from 50 highly malaria endemic districts in the country earmarked by the NVBDCP for an early roll-out of community-based management of malaria by CHWs and population level distribution of LLINs.

### Study design and participants

**Figure 1:**
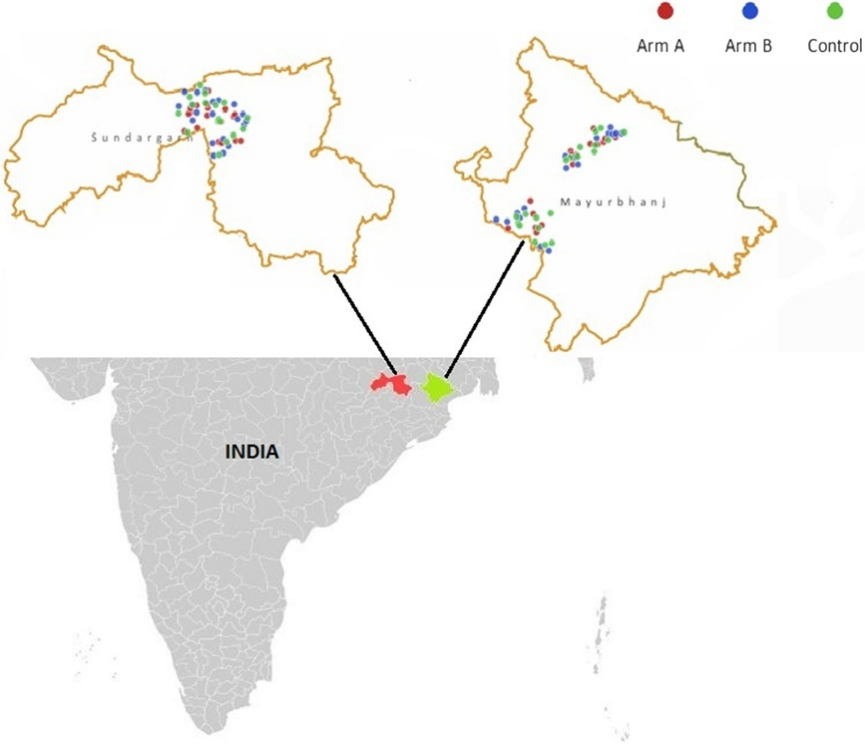
**Location of study villages by treatment status.**

The study consisted of three arms, two arms of intervention –Arms A and B – and one of control. In each study district, two endemic blocks (sub-districts) were randomly selected from among the set of all endemic blocks. In each of the study blocks all endemic villages were enumerated and 10 villages (with an average population of 900) were randomly assigned to arm A, 10 villages randomly assigned to arm B, and 10 villages randomly assigned to observational control. Given the four study sub-districts, the total study population was comprised of 120 villages – 40 in one intervention arm, 40 in another intervention arm, and 40 as controls. The NVBDCP characterizes a village with an annualized parasite incidence (confirmed malaria cases in thousand population per annum) of above five as malaria endemic. Figure 1 presents the geographic distribution of study villages in each of the two districts.

Arm A received supportive supervision of ASHA along with community mobilization support (i.e. combined interventions), while Arm B was provided with only community mobilization activities. The control arm received the routine activities of the government’s malaria control programme, i.e. case management by ASHA without any additional supervision or community mobilization. The routine community mobilization activities in the control villages included two meetings (one each during June and October), one street theatre performance, and one mobile public address campaign with the distribution of informative leaflets on malaria during the year. This study was conceived, implemented, and evaluated in collaboration with the NVBDCP and the Department of Health and Family Welfare (DoHFW), Government of Odisha, which also provided the necessary approval. Ethical approval was obtained from an independent ethical committee in Bhubaneswar, India, which was constituted as per the guidelines of the Indian Council of Medical Research [16].

### Interventions

**Figure 2:**
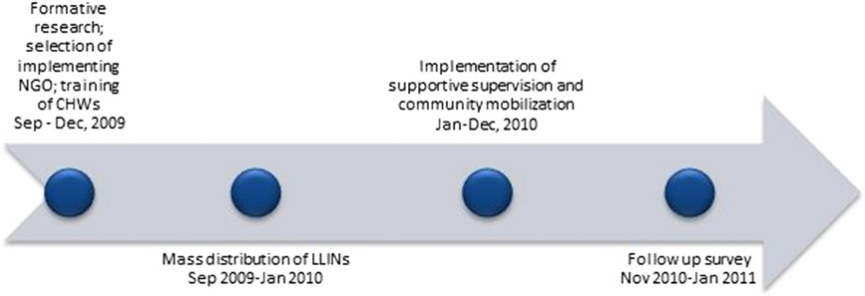
**Timeline of intervention.**

As summarized in project timeline Figure 2, the study was divided into two phases – planning (September-December, 2009) that included formative research, recruitment and training of project staff; and implementation (January-December, 2010) of the interventions. Necessary criteria for NGO participation were the following: (a) previous experience with malaria-related activities and (b) previous activity in the study sub-districts. Only three operating NGOs fulfilled these criteria and were enrolled in the study, two NGOs operating in separate blocks in Sundargarh district while one NGO in Mayurbhanj was able to conduct intervention activities in both blocks. Implementer training conducted by the investigators oriented the participating NGOs on the scope of the project and its effective management. The specific design of the community-based activities and their operationalization required an evidence-base on the communities’ socio-economic and cultural characteristics, life style, health-seeking pattern and knowledge regarding febrile and other common illnesses. Baseline qualitative research provided such evidence [10]. Community level meetings and participatory social mapping exercises conducted in every study village led to further fine-tuning of intervention strategies. These meetings also provided an opportunity for the implementing NGO and the community to build rapport. As part of the national malaria control programme’s strategy, LLINs were distributed and the ASHA were provided with RDT and ACT for management of fever and malaria cases in all three arms. Every study village – both in treatments and control – contained an active ASHA previously trained in malaria case management.

**Figure 3:**
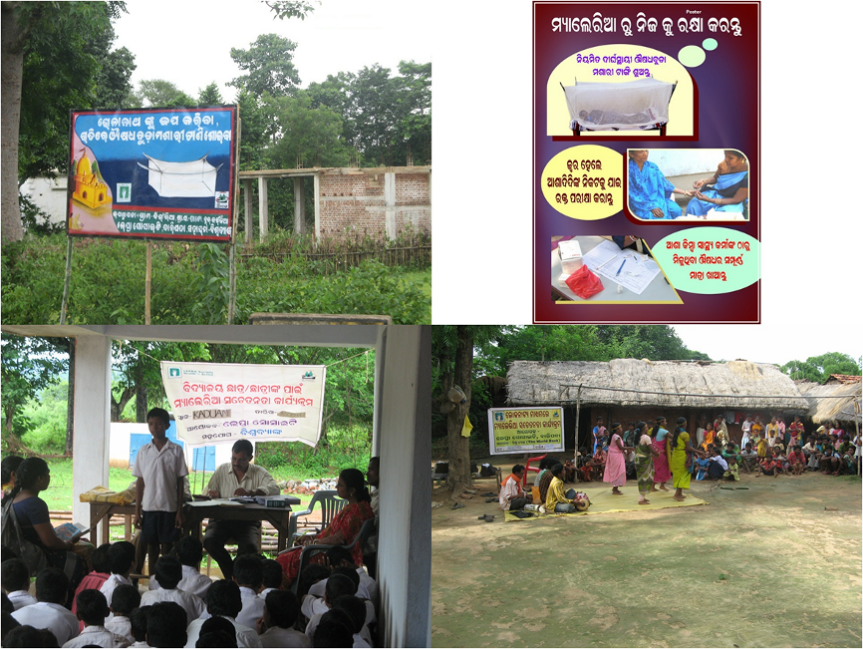
**Sample pictures of community mobilization materials and activities.**

Community mobilization: Community mobilization efforts focused on modifying population health-seeking behaviour towards effective malaria control and management. Specifically, mobilization efforts aimed at 1) increasing the consistent use of long lasting insecticide treated bed nets that were provided to the community free of cost by the NVBDCP, and 2) timely care-seeking for febrile illnesses from the ASHA in the village. Activities included the dissemination of appropriate behaviour change messages through locally acceptable communication channels. The formative research conducted during the planning phase helped incorporate local norms and customs into the design of the community mobilization strategies and messages [10]. Mobilization activities were most intensive during the transmission season with follow up activities afterwards. Various target groups such as local self-government, social organizations, women, men, youth, school and religious groups were chosen for community mobilization. The main messages for the community mobilization activities were as follows: (1) "whenever you have fever, visit the ASHA as early as possible to get your blood tested"; (2) "avail medicines from the ASHA if the blood test is positive for the malaria parasite"; (3) "always consume the full course of drugs given by the ASHA"; (4) "use bed nets every night during sleep"; and (5) "give preference to pregnant women and young children if bed nets are insufficient in the household". The messages were conveyed through community-based meetings (held separately for different target groups considering the local social norms), posters and leaflets, cinema shows, street plays, and community notices (photo examples given in Figure 3). Further, door-to-door visits were undertaken to promote the consistent use of bed nets as well as timely care-seeking from the ASHA for fever. The NGOs utilized local community-based groups (CBO) such as the Village Health and Sanitation Committee (VHSC) and women’s Self Help Groups (SHG) for community mobilization. The SHG members were assigned a few households (10–15) each in every participating village to monitor bed net usage at nights. Details of the community mobilization activities are provided in the Additional file 1.

Supportive supervision: Supportive supervision was designed to improve effective case management of febrile cases by the ASHA by enhancing her professional competence and confidence, increasing community engagement, and ensuring the regular availability of drugs, RDT kits, and other relevant supplies. Under such supervision, a trained NGO field worker visited each ASHA at least twice a month. Every NGO field worker was responsible for 10 ASHA. The supervision activities involved sensitization on the knowledge about transmission, diagnosis and treatment of malaria; hands-on support for performing and interpreting rapid diagnosis tests; administration of the correct dosage of ACT and follow-up to ensure compliance; management of malaria surveillance records; and orientation on community and health centre engagement. District programme managers in conjunction with their local counterparts such as multipurpose health supervisors and workers ensured a regular supply of drugs and commodities at the community level. A typical visit by the NGO field worker lasted for one to two hours for each CHW. In treatment arm A, these activities were conducted in conjunction with the community mobilization activities described above.

### Outcomes

Intervention effectiveness was assessed through a comparison of outcome measures between the intervention and control arms. Main outcome measures were related to the reported consistent usage of LLINs and care-seeking patterns of febrile cases. Specific measures included proportion of fever cases-seeking care from a trained provider and receipt of test and treatment, if appropriate, for malaria within a day of developing symptoms; households owning at least one LLIN; and population sleeping under a bed net.

### Evaluation

A brief quantitative household survey instrument was implemented in 90 study villages before intervention activities and a more extensive household and community survey was conducted in all study villages at the end of the intervention period (November 2010-January 2011). Study instruments utilized the local language Odia and were piloted and modified before each survey. The baseline survey collected basic demographic data from 22 households per village. These data are mainly used to explore balance of socio-demographic characteristics across intervention arms.

For the end line survey, instruments consisted of a household questionnaire and an individual-level questionnaire administered to recent (two-week recall) fever cases. The household-level questionnaire recorded demographic, socio-economic and health characteristics, general health-seeking behaviour, knowledge on malaria and utilization of bed nets. The individual fever questionnaire collected information on treatment-seeking behaviour from the recent fever cases. In each study village, a full household listing was conducted from which 10 randomly selected households were interviewed for the household level information. The full household listing also included a listing of all recent fever cases (determined through two-week recall) and 10 cases were randomly selected from each village and interviewed for individual-level information. For both surveys, interviews were recorded on paper forms and double-entered in CS Pro software (version 4.0) at a central location. Project level cost data were extracted from the financial reports and government level data from the registers at the health centres.

For the sample size estimates, two major outcomes of interest were considered, i.e. proportion of fever cases tested for falciparum malaria within 24 hours and proportion of households correctly utilizing at least one LLIN. The rate of fever cases tested within 24 hours of onset of symptoms was assumed to be 35% at the baseline. With 40 clusters in each arm and at an intra-cluster correlation estimate of 0.1, the study is sufficiently powered (significance of 0.05 and power of 0.8) to identify an increase in the prompt fever testing rate of 21 percentage points. Similarly, the study is sufficiently powered (significance of 0.05 and power of 0.8) to detect an increase in net utilization from 35% to 51% at an intra-cluster correlation estimate of 0.1.

### Statistical analysis

The data were analysed as an intention-to-treat analysis with treatment at the cluster (village) level. Balance across treatment arms in pre-intervention or fixed characteristics measured at end-line but unaffected by the intervention were assessed through normalized mean differences and differences exceeding a threshold of 25% were considered significant [21]. Pair-wise t-tests of difference were also estimated. Differences in outcomes between intervention and control clusters were examined with logistic regression. Socio-economic status (SES) was calculated by a principal component analysis of key household characteristics and assets to create a wealth index [22]. Since no differences were found between unadjusted and adjusted odds ratios – i.e. results are unchanged if adjusted for the observable characteristics. Thus, all odds ratios presented below are unadjusted. Typically, with clustered outcomes such as here, robust standard errors adjusted for clustering at the village level are reported [23]; however, given that only binary response outcomes are analysed with logistic regression, clustered standard errors are identical to unclustered standard errors. Data were analysed with Stata software (version 12).

Cost data were calculated on the expenditures for each type of intervention consisting of human resources (including time, travel and per diems), training, community mobilization, stationery and overheads. The costs were compared with the outcomes (i.e. bed net use and timely treatment-seeking) extrapolated at the population level for the study clusters. Incremental cost effectiveness ratios were estimated against the control arm.

## Results

### Balance of key characteristics across treatment arms

As village randomization into treatment or control was conducted before the collection of population information, successful randomized assignment is checked through a comparison of potentially influential population characteristics across treatment and control arms that may influence the outcomes of interest. Tables 1 and 2 present, respectively, the baseline and endline means of such characteristics in the three study arms as well as the normalized mean difference for each pair-wise comparison across study arms. Randomized assignment appears to have resulted in a balanced study sample across a wide range of population characteristics. Only one standardized mean-difference exceeds the 25 percent threshold [21]; even that mean difference, API at baseline between arms B and control, is only at 25.5 percent. Any observed differences in intervention performance are unlikely to have been driven by an imbalance of characteristics across treatment arms as virtually none is observed. Next, unadjusted odds ratios are used to measure programme impact on targeted outcomes such as bed net ownership and utilization, fever care-seeking behaviour, and village-level fever prevalence.

**Table 1:** **Endline mean characteristics in intervention and control clusters, and normalized mean differences across study arms** ^**#** * +^

	Supportive supervision and community mobilization (Arm A)	Community mobilization (Arm B)	Control	Normalized differences: Arms A-B	Normalized differences: Arms A-K	Normalized differences: Arms B-K
Annual malaria parasite incidence per cluster	12.26	10.79	9.12	-0.025	-0.049	0.255
**Household characteristics n/N (%)**						
Hindu	304/390 (77.9)	291/400 (72.8)	298/390 (76.4)	0.085	0.026	-0.058
Christian	74/390 (18.9)	96/400 (24.0)	78/390 (20.0)	-0.088	-0.020	0.068
Others	12/390 (3.1)	13/400 (3.3)	14/390 (3.6)	-0.008	-0.020	-0.012
Scheduled Tribe	282/390 (72.3)	306/400 (76.5)	303/390 (77.7)	-0.068	-0.088	-0.020
Scheduled Caste	26/390 (6.7)	34/400 (8.5)	21/390 (5.4)	-0.048	0.039	0.086
Others	82/390 (21.0)	60/400 (15.0)	66/390 (16.9)	0.159	0.109	-0.036

^#^None of the 21 pairwise t-tests for equality of means across study arms revealed a significant difference between the average household characteristics, at a 5% level of significance.

*There are 390 households in Arm A, 400 in Arm B and 390 in the control arm.

**Table 2:** **Baseline mean characteristics in intervention and control clusters, and normalized mean differences across arms** ^# *^

	Supportive supervision and community mobilization (Arm A)	Community mobilization (Arm B)	Control	Normalized differences: Arms A-B	Normalized differences: Arms A-K	Normalized differences: Arms B-K
Wealth Index	0.452 (0.696)	0.372 (0.628)	0.337 (0.611)	0.085	0.124	0.040
Livestock (count)	2.131 (2.478)	2.413 (2.953)	2.362 (2.824)	-0.073	-0.061	0.012
Poultry (count)	4.926 (6.836)	4.885 (7.624)	5.095 (6.633)	0.004	-0.018	-0.021
Cropped During Previous Season (proportion)	0.982 (0.133)	0.985 (0.123)	0.983 (0.131)	-0.017	-0.005	0.011
Household Has Bank Account (proportion)	0.810 (0.393)	0.803 (0.399)	0.777 (0.417)	0.012	0.058	0.045
Household Head is Male (proportion)	0.913 (0.282)	0.910 (0.287)	0.918 (0.275)	0.007	-0.013	-0.020
Household Head is Currently Married (Proportion)	0.885 (0.320)	0.848 (0.360)	0.867 (0.340)	0.077	0.039	-0.038
Household Head Has Less Than Primary Education (proportion)	0.309 (0.463)	0.307 (0.462)	0.290 (0.455)	0.003	0.029	0.026
Males in Wage Labor (count)	0.730 (0.444)	0.773 (0.419)	0.805 (0.397)	0.070	-0.126	-0.055
Females in Wage Labor (count)	0.415 (0.493)	0.473 (0.500)	0.541 (0.499)	-0.083	-0.180	-0.096
Household Has Non-farm Enterprise (proportion)	0.200 (0.401)	0.258 (0.438)	0.167 (0.373)	-0.098	0.060	0.158
Household Younger than 5 (proportion of total)	0.101 (0.132)	0.109 (0.142)	0.112 (0.146)	-0.041	-0.056	-0.015
Total Household Size (count)	5.500 (2.100)	5.458 (2.188)	5.359 (1.870)	0.014	0.050	0.034

^#^None of the 39 pairwise t-tests for equality of means across study arms revealed a significant difference between the average household characteristics at a 5% level of significance.

^*^There are 788 households in Arm A, 781 in Arm B and 775 in the control arm.

^+^Standard deviations in parentheses.

### Effects on preventive malaria related behaviours

Almost all households in the study sample (99%) owned at least one bed net (Table 3). This lack of significant difference across study arms is not surprising since all three received wide distribution of free LLINs. However, bed net use patterns show more variation across study arms. Significantly more respondents reported to have slept under a bed net the previous night of the survey in Arm A (84.54%; p < 0.001; 95% CI 1.328-1.661) and Arm B (82.43%; p < 0.001; 95% CI 1.143-1.419) than the control arm (78.65%). Almost 97 percent of all children in arm A (p = 0.003; 95% CI 1.383-4.688) and 94% in arm B (p = 0.01; 95% CI 1.186-3.592) slept under a bet net, while it was less than 91 percent in the control arm. Women of reproductive age in arm A reported significantly higher use of bed net than the control arm (96.79% vs. 94.09; p = 0.006).

**Table 3:** **Reported utilization of bed nets by intervention arm and relative odds ratios of intervention impacts**

	Supportive supervision + community mobilization	Community mobilization	Control	Supportive supervision + community mobilization versus control		Community mobilization versus control	
	n/N (%)	n/N (%)	n/N (%)	Odds ratio (95% CI)	p value	Odds ratio (95% CI)	p value
**Bed net ownership**							
Households with at least one bed net	760/768 (99.15)	774/781 (99.1)	750/755 (99.34)	0.633 [0.206, 1.945]	0.425	0.737 [0.233, 2.33]	0.604
**Slept last night under a bed net**							
Total population	3,571/4,224 (84.54)	3,589/4,354 (82.43)	3,219/4,093 (78.65)	1.485 [1.328, 1.661]	0.000	1.274 [1.143, 1.419]	0.000
Children under 5 years	451/466 (96.78)	488/508 (94.29)	461/500 (90.68)	2.544 [1.383, 4.688]	0.003	2.064 [1.186, 3.592]	0.010
Women of Childbearing Age (15–49 years)	998/1,031 (96.79)	990/1,035 (95.65)	934/991 (94.09)	1.846 [1.191, 2.859]	0.006	1.343 [0.899, 2.005]	0.149

### Effects on care-seeking behaviour for fever

Diagnosis and treatment within 24 hours are crucial to decreasing morbidity and mortality from malaria. Providers were considered as trained if they had been trained by the malaria control programme, including medical doctors, nurses, multipurpose health workers and CHWs. Table 4 shows that diagnosis within a day of the onset of fever was not significantly different between the intervention and control arms for any study sub-group. However prompt diagnosis from a trained provider is significantly higher in both intervention arms (60.6%; OR = 1.529; p = 0.004 and 59.3%; OR = 1.450; p = 0.007 vs. 50.1% in control). This effect is even more pronounced when restricting the analysis to young children (63.2%; OR = 1.935; p = 0.059 and 63.51%; OR = 1.958; p = 0.049 vs. 47.1% in control) or women of reproductive age (61.6%; OR = 1.867; p = 0.028 and 64.3%; OR = 2.094; p = 0.006 vs. 47.2% in control). Further, both interventions shifted care-seeking towards front-line representatives – diagnosis from a CHW was significantly higher in both intervention arms (28%; OR = 1.642; p = 0.005 and 27.6%; OR = 1.603; p = 0.007) than in the control arm (19.2%). Focusing on CHW performance, proportionately more fever cases visiting an ASHA in Arm A had timely diagnosis than the control arm (82.08% *vs* 67.14%; OR = 2.24; p = 0.025).

**Table 4:** **Reported fever care-seeking and treatment behaviour by intervention arm**

	Supportive supervision + community mobilization	Community mobilization	Control	Supportive supervision + community mobilization versus control		Community mobilization versus control	
	n/N (%)	n/N (%)	n/N (%)	Odds ratio (95% CI)	p value	Odds ratio (95% CI)	p value
**Prompt fever diagnosis (<24 hrs)**							
Total fever cases	261/378 (69.05)	260/381 (68.24)	248/365 (67.95)	1.05 [0.772, 1.434]	0.746	1.014 [0.745, 1.379]	0.931
Children under 5 years	46/68 (67.65)	54/74 (72.97)	42/68 (61.76)	0.773 [0.382, 1.564]	0.473`	0.598 [0.295, 1.22]	0.156
Women	71/99 (71.72)	81/126 (64.29)	65/106 (61.32)	1.054 [0.777, 1.429]	0.736	1.106 [0.814, 1.501]	0.520
**Prompt fever diagnosis (<24 hrs) by a trained provider**							
Total	229/378 (60.58)	226/381 (59.32)	183/365 (50.14)	1.529 [1.143, 2.045]	0.004	1.450 [1.086, 1.937]	0.012
Children under 5 years	43/68 (63.24)	47/74 (63.51)	32/68 (47.06)	1.935 [0.975, 3.840]	0.059	1.958 [1.001, 3.832]	0.049
Women	61/99 (61.61)	81/126 (64.29)	49/106 (47.22)	1.867 [1.070, 3.258]	0.028	2.094 [1.235, 3.549]	0.006
**Fever diagnosed by a CHW**							
Total	106/378 (28.04)	105/381 (27.56)	70/365 (19.18)	1.642 [1.164, 2.316]	0.005	1.603 [1.114, 2.262]	0.007
Children under 5 years	13/53 (24.53)	12/55 (21.82)	9/53 (16.98)	1.589 [0.614, 4.115]	0.340	1.364 [0.522, 3.567]	0.526
Women	29/99 (29.29)	34/126 (26.98)	20/106 (18.87)	1.782 [0.929, 3.417]	0.082	1.589 [0.850, 2.971]	0.147
**Prompt (<24 hrs) fever diagnosis by a CHW**							
Total	87/106 (82.08)	83/105 (79.05)	47/70 (67.14)	2.241 [1.108, 4.529]	0.025	1.846 [0.930, 3.664]	0.080
Children under 5 years	12/13 (92.31)	9/12 (75.00)	6/9 (66.67)	7.549 [0.509, 70.668]	0.154	1.500 [0.223, 10.077]	0.677
Women	24/29 (82.76)	26/34 (76.47)	16/20 (80.00)	1.2 [0.279, 5.162]	0.807	1.846 [0.930, 3.664]	0.080
**Fever treatment by provider**							
Community Health Worker	106/378 (28.04)	105/381 (27.56)	70/365 (19.18)	1.642 [1.164, 2.316]	0.005	1.603 [1.137, 2.617]	0.007
Other trained providers	43/378 (11.38)	44/381 (11.55)	29/365 (7.95)	1.487 [0.907, 2.439]	0.116	1.513 [0.924, 2.476]	0.100
Medical Doctors	161/378 (42.59)	154/381 (40.42)	164/365 (44.93)	0.909 [0.680, 1.215]	0.521	0.832 [0.622, 1.112]	0.213
Untrained providers	41/378 (10.85)	52/381 (13.65)	77/365 (21.10)	0.455 [0.302, 0.686]	0.000	0.591 [0.402, 0.869]	0.008
No treatment sought	27/378 (7.14)	26/381 (6.82)	25/365 (6.85)	1.046 [0.595, 1.839]	0.875	0.996 [0.564, 1.759]	0.989
**Prompt (<24 hrs) fever treatment**							
Total	236/378 (62.44)	226/381 (59.32)	190/365 (52.06)	1.530 [1.143, 2.051]	0.004	1.343 [1.005, 1.794]	0.046
Children under 5 years	48/71 (67.61)	47/74 (63.51)	35/68 (51.47)	1.968 [0.989, 3.915]	0.054	1.641 [0.839, 3.211]	0.148
Women	61/99 (61.61)	67/126 (53.18)	50/106 (47.17)	1.798 [1.031, 3.136]	0.039	1.272 [0.758, 2.134]	0.363
**Prompt (<24 hrs) fever treatment by a trained provider**							
Total	229/378 (60.58)	226/381 (59.32)	183/365 (50.14)	1.529 [1.143, 2.045]	0.004	1.450 [1.086, 1.937]	0.012
Children under 5 years	43/71 (63.24)	47/74 (63.51)	32/68 (47.06)	1.935 [0.975, 3.840]	0.059	1.958 [1.001 3.832]	0.050
Women	61/99 (61.62)	67/126 (54.03)	49/106 (47.12)	1.802 [1.030, 3.151]	0.039	1.319 [0.783, 2.224]	0.298

The survey also asked about the receipt of any malaria treatment. Treatment from any kind of trained providers was more prevalent in the intervention arms; some of this change came from substitution away from untrained providers (10.85% in arm A, 13.65 percent in arm B, 21.1% in control). Further, significantly more fever cases from both arm A (60.58%; OR = 1.529; p = 0.004) and arm B (59.32%; OR = 1.45; p = 0.012) than controls (50.14%) received timely treatment from a trained provider. In particular, women from arm A were more likely than women in control areas to receive prompt treatment from a trained provider (61.62% vs. 47.12%; OR = 1.8; p = 0.039). Overall timely treatment-seeking was found to be higher in treatment areas. However, these results were not statistically significant.

### Effects on reported fever incidence

It was examined whether changes in bed net use and fever care-seeking patterns resulted in decreases in the village-level incidence of malaria or other febrile illness. Using estimated community rates of two-week fever incidence during the high transmission period, reported fever incidence in treatment villages was found to be lower than the control villages: 15.5% in both Arms A and B relative to 17.7% in control; however, these differences were not statistically significant (p-values of 0.16 and 0.20 respectively).

### Cost effectiveness analysis

The per capita cost of the combined interventions was 97 US cents and community mobilization was 62 cents, whereas the routine programme cost 10 cents. The incremental cost for combined interventions was $13.07 per additional person reported to sleep under a bed net the night before the survey, whereas it was $14.26 for community mobilization. The combined interventions arm was more effective at increasing bed net use, timely diagnosis by a trained provider, and timely treatment by a CHW, while the community mobilization arm was more cost-effective at improving timely diagnosis by a CHW and timely treatment by a trained provider. The details about the cost-effectiveness analysis are provided in the Additional file 2.

## Discussion

A community-based intervention targeting prevention and management of malaria in Odisha, India, attempted to (1) empower CHWs with training and support; (2) utilize intensive community mobilization with reliance on the traditional media considering the local social and cultural norms; (3) build local capacity through community based organizations and groups to enhance the effectiveness of malaria case management by CHWs; and (4) demonstrate a public sector programme model of partnership between the public sector, private not-for-profit sector, and the community to enhance sustainability. These interventions led to significant improvements in reported bed net use, especially for vulnerable sub-groups, and timely care-seeking from a trained health care provider. Results show significant increases in the reported *utilization* of bed nets in treatment arms relative to controls, which is particularly encouraging because the surveys were conducted towards the end of the high transmission season and there were no significant differences in the *ownership* of bed nets between households in treated and control villages. The increases in utilization were somewhat more pronounced among the villages where community mobilization was supplemented with supportive supervision of the community health workers.

The studied intervention sought to strengthen the Indian CHW (ASHA) programme through supportive supervision. While the ASHA have been integrated into the national malaria control programme, they are female volunteers with only primary education, selected by the rural communities they reside in, and do not have any formal training in healthcare prior to their selection. Their low levels of formal education and lack of experience with the health sector suggests the potential for hands-on support of specific management of diseases and health conditions. This study demonstrates that a supportive intervention on malaria case management by CHWs shifted care-seeking behaviour and bed net use in desirable ways in two highly malaria endemic districts. The supportive supervision by NGO workers through semi-monthly visits provided them with a structured learning process. Similar to other low- and middle-income country settings, more hands-on support through supportive supervision imparted more confidence, knowledge and skills in CHWs and thereby improved their motivation to perform [13–16]. Further, the supervisors provided the conduit for efficient communication between the CHWs and the formal health system to maintain an uninterrupted supply of commodities. Through supportive supervision, the study brought in considerable change in the community’s acceptance and response towards CHWs in contrast to the situation in control communities [10]. Indeed, in a particularly encouraging sign, treated households moved away from seeking fever care from untrained providers to the ASHA. Interestingly, other trained providers also noticed a drop in the proportion of total cases compared to the control villages due to the care-seeking from the CHWs, which may benefit the health system by allowing more prompt diagnosis and treatment of fever and by letting trained providers devote their time and skills to the management of more complicated health conditions as CHWs deal with uncomplicated fever cases at the village level in a cost-effective manner [24].

This shift in care-seeking from facility based providers to community health workers is consistent with patterns observed from similar supportive supervision interventions in malaria endemic settings in Africa [25–29]. Since malaria is typically endemic in remote areas with hilly terrain, a tailored community health worker or volunteer model may be most suitable for disease control and management. However, care should be taken to ensure that the supervisors are adequately oriented and skilled on key aspects of malaria control and management of community health.

The intervention introduced globally proven methods (RDT, ACT and LLIN) with locally adapted delivery strategies to achieve the targets of "Roll Back Malaria" for women and children under five [2, 12]. The targeted vulnerable populations of children under-five and women of childbearing age benefitted in particular from a greater utilization of both bed nets and fever care services. The impact on these vulnerable populations could be an effect of the enhanced case management activities by the CHW, who was a female from the same village with an in-depth understanding of the socio-cultural context. The involvement of women’s groups in the intervention may have further facilitated prompt care-seeking among women and children, although the present study is unable to explicitly test this channel of impact. The deployment of female CHWs and women's groups in community health management is likely reflected in terms of community health awareness and behaviour [30–33]. The community’s health-seeking pattern for fever distinctly shifted from untrained to trained providers, which suggests the potential for minimizing inappropriate treatment regimens, catastrophic health expenses and consequent fatalities [3, 10]. These findings are consistent with the evidence from similar Asian and African settings about leveraging local capacity to ensure sustainability of community health approaches [34]. The thrust of the intervention was to identify and empower local stakeholders especially CBOs and women’s groups on building up social trust, cohesion, support, mutual capacity building and thereby improving positive health-seeking behaviour [35, 36]. Locally constituted women’s groups are well-poised to be cost-effective and sustainable change makers for community mobilization and gradual behaviour changes [30, 31, 33].

The studied intervention identified and built local capacity to enhance the effectiveness of malaria case management by CHWs and demonstrated a model for locally sustainable community based service-delivery and monitoring. The community mobilization relied on the traditional media and involved various community structures considering the social and cultural norms. The design and dissemination of the community mobilization strategy were based on a bottom-up approach with the participation of the community. Apart from engaging women’s groups, the intervention also capitalized on other community-level formal and informal associations, such as local self-government, village health and sanitation committees, men’s groups and youth clubs. Print and electronic media supplemented the group activities and community notices and the interventions were intensively aligned with the disease transmission season to maximize impact. Empowerment of community entities is a corner stone of the community focus for public health interventions and is also a mandate of India’s National Rural Health Mission [37]. However, community based organization for supportive supervision and management must be carefully chosen to be locally acceptable and possess adequate coordinating capacity. Transparency, clear delegation of responsibilities and coordination among various stakeholders, including CHWs, is essential to the success of such interventions. As the project suggests, linking the CHW with the higher levels of the health facilities to ensure uninterrupted supply of commodities, recording of health information and monitoring, is another key component of the potential success of such supportive interventions.

This project introduced a three-way partnership between the public sector, private not-for-profit sector, and the community, i.e. public-private-community participation (PPCP). The engagement of local NGOs enabled the easy rollout and monitoring of the project, allowed the intervention to be incorporated into the public sector programme, and led to sustained activities rather than duplicating or substituting for any pre-existing programme activity. However, as may be expected with such community mobilization interventions, the cost of implementation was high in our interventions compared to the standard programme. Note, however, that the total cost of the combined intervention was 97 cents per capita, which is slightly lower than the $1.06 per capita cost of similar a community mobilization programme involving shopkeepers and communities in rural Kenya [38]. The fixed nature of start-up and administration costs will further decrease the cost of this intervention if it is implemented over a longer period. As the community becomes more aware of the malaria control activities and changes its health-seeking behaviour, the intensity of the community mobilization activities could be scaled down, further bringing down total costs.

This study is not without limitations. In traditional rural Indian settings, informal sharing of information is common among the inhabitants of a locality; thus, informational spillovers might have contaminated the control group particularly since the treatment and control villages were often geographically contiguous villages. However, outcomes in neighbouring treated villages (weighted by distance to the treated village) do not seem to have a significant impact on outcomes in control villages. This lack of a significant relationship suggests that the results reported above are not contaminated by spillovers. Note that even if spillovers existed, they would have led to a downward bias in the estimated treatment effect since such spillovers would have improved outcomes in control areas. Secondly, while recall bias is not uncommon in community-based surveys, any such bias would have influenced all three study arms in a similar manner. Finally, self-reported preventive behaviour may have been biased by social desirability concerns. This type of reporting bias has been observed when contrasting behaviour recorded at the health facility and data reported through household survey, with survey data presumed to be the more accurate [39, 40]. Differential programme effectiveness observed by district suggests that desirability bias cannot fully account for the programme impacts measured here as certain implementers are more effective in achieving outcomes [41]. Nevertheless, any such reporting bias may results in an overestimation of programme effects for self-reported preventive behaviours.

## Electronic supplementary material

Additional file 1: **Summary of community mobilization activities conducted in both treatment arms.** Description of community mobilization interventions (frequency and methods). (DOCX 14 KB)

Additional file 2: **Cost effectiveness analysis. Detailed description of the cost effectiveness analysis of the intervention.** (DOCX 19 KB)
