# Supplementary material for: Strengthening malaria service delivery through supportive supervision and community mobilization in an endemic Indian setting: an evaluation of nested delivery models
Source: Malar J. 2014 Dec 8;13:482. doi: 10.1186/1475-2875-13-482 (PMC4320454; doi:10.1186/1475-2875-13-482)
Supplement: Supplementary file 1 — Additional file 1: Summary of community mobilization activities conducted in both treatment arms. Description of community mobilization interventions (frequency and methods). (DOCX 14 KB) [file 12936_2014_3685_MOESM1_ESM.docx]

| **Method** | **Frequency** |
| --- | --- |
| Community hoarding (billboards) | One in each village |
| Community meetings | Twice a year each separately for men’s groups, women’s groups, village health and sanitation committees, churches |
| Flip book | Distributed during the community meetings once a year |
| Community based organization booklet | Distributed during the community meetings once a year |
| School meetings | Twice a year |
| School booklet and malaria wheel | Distributed during the schools meetings twice a year |
| Folk media (street play) | Twice a year |
| Audio-visual show | Twice a year |
| Posters and leaflets | Distributed during street play and audio-visual show, community meetings |

**Additional file 1**

**Summary community mobilization activities conducted in both treatment arms**
