# Supplementary material for: Strengthening malaria service delivery through supportive supervision and community mobilization in an endemic Indian setting: an evaluation of nested delivery models
Source: Malar J. 2014 Dec 8;13:482. doi: 10.1186/1475-2875-13-482 (PMC4320454; doi:10.1186/1475-2875-13-482)
Supplement: Supplementary file 2 — Additional file 2: Cost effectiveness analysis. Detailed description of the cost effectiveness analysis of the intervention. (DOCX 19 KB) [file 12936_2014_3685_MOESM2_ESM.docx]

**Additional file 2**

**Cost effectiveness analysis**

Cost data were calculated from a provider’s perspective for each type of intervention consisting of human resources (including time, travel and per diems), training, community mobilization, stationery and overheads. Total cost was divided by the population for the study area to compute per capita cost of the interventions. The costs were compared with the outcomes (i.e. bed net use and timely treatment seeking) extrapolated at the population level for the study clusters. The effectiveness of the interventions were defined by the gains in the outcomes, e.g. additional people sleeping under the bed net compared to the standard program. Incremental cost effectiveness ratios (ICER) were calculated by dividing the differences in cost between intervention and control (incremental cost) with the differences in the outcomes between intervention and control (incremental effectiveness) as shown by the formula below. 𝐼𝐶𝐸𝑅=𝐶𝑜𝑠𝑡𝐼𝑛𝑡𝑒𝑟𝑣𝑒𝑛𝑡𝑖𝑜𝑛−𝐶𝑜𝑠𝑡𝐶𝑜𝑛𝑡𝑟𝑜𝑙𝑂𝑢𝑡𝑐𝑜𝑚𝑒𝐼𝑛𝑡𝑒𝑟𝑣𝑒𝑛𝑡𝑖𝑜𝑛−𝑂𝑢𝑡𝑐𝑜𝑚𝑒𝐶𝑜𝑛𝑡𝑟𝑜𝑙

Two assumptions were made while doing the cost analysis. First, all members of the community were exposed equally to the interventions; second, for each assessed outcome, the amount was entirely spent on that particular outcome. No discounting was applied as the project was implemented for only a year. The mean exchange rate for the US dollar was 45.75 Indian Rupees (1 INR = 2 US Cents approx.) during the study period.

We compared the costs and outcomes between supportive supervision and community mobilization with the control arm as the base case. The per capita cost of the combined interventions was 97 US cents and community mobilization was 62 cents, whereas the routine program cost 10 cents (table A1). Applying the proportion of people sleeping under bed nets from the survey population (84.54% Arm A, 82.43% Arm B and 78.65% control) to the total population in the intervention area, we extrapolate that 38227 people sleeping under a bed net was estimated in Arm A, 37273 in Arm B, and 35564 in the control arm. Hence, relative to the control arm, 2663additional people slept under a bed net in Arm A and 1709 in Arm B. The combined interventions (Arm A) would cost $13.07 per additional person reported to sleep under a bed net the night before the survey, whereas only community mobilization (Arm B) would cost $14.26. We applied similar principles to estimate the incremental effectiveness of other outcome indicators on timely diagnosis and treatment. Between the two interventions, the combined interventions arm was thus most effective at increasing bed net use, timely diagnosis by a trained provider, and timely treatment by a CHW. Community mobilization, on the other hand was cost-effective at improving timely diagnosis by a CHW and timely treatment by a trained provider.

**Cost-effectiveness analysis of the interventions**

|  | **Supportive supervision and community mobilization** | **Community mobilization** | **Standard program** |
| --- | --- | --- | --- |
| Population coverage [A] | 39645 | 45218 | 34402 |
| Total cost of intervention (USD) [B] | 38388 | 27959 | 3584 |
| Per capita cost (USD) [B/A] | 0.97 | 0.62 | 0.10 |
| Incremental cost with control as the base (USD) [C] | 34803.96 | 24375.11 | CG |
| Proportion of survey sample sleeping under a bednet (%) [D] | 84.54 | 82.43 | 78.65 |
| Estimated number sleeping under a bed net [E = D*A] | 38227 | 37273 | 35564 |
| Additional people sleeping under a bed net with control as the base [F] | 2663 | 1709 | CG |
| Incremental cost-effectiveness ratio for bed net use (USD) [C/F] | 13.07 | 14.26 | CG |
| Estimated fever cases [G] | 1018 | 1018 | 1017 |
| Proportion of survey sample timely diagnosis by a CHW (%) [H] | 82.08 | 79.05 | 67.14 |
| Estimated fever cases diagnosed timely by a CHW [I = H*G] | 836 | 805 | 683 |
| Additional fever cases diagnosed timely by a CHW with control as the base [J] | 153 | 122 | CG |
| Incremental cost-effectiveness ratio for timely diagnosis by a CHW (USD) [C/J] | 227.48 | 199.80 | CG |
| Proportion of survey fever sample timely diagnosis by a trained provider (%) [K] | 53.87 | 50.92 | 44 |
| Estimated fever cases diagnosed timely by a trained provider  [L = K*G] | 549 | 519 | 450 |
| Additional fever cases diagnosed timely by a trained provider with control as the base [M] | 98 | 68 | CG |
| Incremental cost-effectiveness ratio for timely diagnosis by a trained provider (USD) [C/M] | 355.14 | 358.46 | CG |
| Proportion of survey fever sample timely treated by a CHW (%) [N] | 21.4 | 12.3 | 2.7 |
| Estimated fever cases timely treated by a CHW [O = N*G] | 218 | 126 | 27 |
| Additional fever cases timely treated by a CHW with control as the base [P] | 191 | 81 | CG |
| Incremental cost-effectiveness ratio for timely treatment by a CHW (USD) [C/P] | 182.22 | 300.93 | CG |
| Proportion of survey fever sample timely treated by a trained provider (%) [Q] | 60.82 | 59.32 | 51 |
| Estimated fever cases timely treated by a trained provider [R = Q*G] | 619 | 604 | 515 |
| Additional fever cases timely treated by a trained provider with control as the base [S] | 104 | 89 | CG |
| Incremental cost-effectiveness ratio for timely treatment by a trained provider (USD) [C/S] | 334.34 | 274.65 | CG |

ICER - Incremental cost-effectiveness ratio; CG – Comparison group
